# Adjuvanted RNA Origami—A Tunable Peptide Assembly Platform for Constructing Cancer Nanovaccines

**DOI:** 10.3390/vaccines13060560

**Published:** 2025-05-25

**Authors:** Theresa Yip, Xinyi Tu, Xiaodong Qi, Hao Yan, Yung Chang

**Affiliations:** 1 School of Life Sciences, Arizona State University, Tempe, AZ 85281, USA; thyip@asu.edu; 2 Biodesign Center for Molecular Design and Biomimetics, Biodesign Institute, Arizona State University, Tempe, AZ 85281, USA; xinyitu@asu.edu (X.T.); xiaodong@asu.edu (X.Q.); 3 School of Molecular Sciences, Arizona State University, Tempe, AZ 85281, USA

**Keywords:** cross-presentation, melanoma, peptide vaccine, polylysine-linked peptide, RNA origami nanostructure, T cell immunity

## Abstract

Background/Objectives: Cancer peptide vaccines represent a promising strategy to develop targeted and personalized treatments for cancer patients. While tumor peptides alone are insufficient in mounting effective immune responses, the addition of adjuvants can enhance their immunogenicity. Nanoparticle delivery systems have been explored as vaccine carriers to incorporate both adjuvants and peptides. One such nanoparticle is RNA origami (RNA-OG), a nucleic acid nanostructure that is programmed to form different sizes and shapes. Our designed RNA-OG can incorporate various biomolecules and has intrinsic adjuvant activity by acting as a toll-like receptor 3 agonist. We previously showed that the RNA-OG functions as an adjuvanted, carrier-free vaccine platform to assemble peptides. Although effective, only a fixed number of peptides (13) could be covalently linked to each RNA-OG. Methods: Here, we developed a simple physical assembly strategy to attach polylysine-linked neopeptides onto RNA-OG so that the number of peptides per RNA-OG could be readily tuned and tested for their immunogenicity. Results: Although the vaccines with a high number of peptides, i.e., 100–200 peptides/RNA-OG, led to greater peptide presentation by bone marrow-derived dendritic cells, they failed to mount effective CD8^+^ T cell responses against engrafted tumor cells, probably owing to an induction of early T cell exhaustion. Interestingly, the same vaccine format with a low number of peptides, i.e., 10–15 peptides/RNA-OG, enhanced CD8^+^ T cell responses without provoking T cell exhaustion in tumor-bearing mice, leading to strong protective anti-tumor immunity. In comparison, the covalently assembled RNA-OG-peptide vaccine, having a similarly low peptide dosage, offered the highest therapeutic efficacy. Thus, our RNA-OG nanostructure provides a simple and tunable platform for peptide loading to optimize vaccine efficacy. Conclusions: Our findings have significant implications for peptide vaccine design regarding peptide dosages and structural stability of RNA-OG complexed with peptides, which could guide the development of more effective peptide vaccines for cancer immunotherapy.

## 1. Introduction

The success of cancer immunotherapy in recent years has promoted research into developing more robust and personalized treatments [1,2]. These immunotherapies aim to prime the host to elicit specific immunity against tumor cells while minimizing any potential adversity to normal cells. Cancer peptide vaccines, comprised of neoepitopes presented by MHC-I, are a promising approach, as they elicit tumor-targeted, cell-mediated responses by mobilizing tumor-specific CD8^+^ T cells to eliminate tumor cells [3]. Although tumor peptides alone are insufficient in producing such responses [4,5], the addition of adjuvants, such as synthetic pathogen-associated molecular patterns (PAMPs) that are sensed by pattern recognition receptors (PRRs) in innate immune cells, can enhance the immunogenicity of the peptides. These adjuvants activate antigen-presenting cells (APCs), especially dendritic cells (DCs), by upregulating the expression of co-stimulatory molecules, such as CD80 and CD86 [6]. The presence of both signals—MHC-I/peptide and co-stimulators by DCs—is required for CD8^+^ T cell activation [7]. However, systemic delivery of potent adjuvants often causes toxicity [8,9,10]. Thus, both the efficacy and safety of adjuvants must be considered when incorporating them into peptide vaccine designs.

Nanoparticle delivery systems, such as lipid and polymer nanoparticles, have been explored for packaging antigens and adjuvants. The encapsulation of these two components prevents systemic entry of free peptides and adjuvants and instead directs them to the draining lymph nodes (LNs), where they can be taken up by DCs, providing safe and efficient delivery [10,11,12]. Furthermore, attachment of the peptides to nanoparticles prevents direct binding of free peptides to MHC-I, which are expressed by all nucleated cells, including those that fail to act as APCs. The peptides carried by nanoparticles could be cross-presented by DCs, a process by which external antigens are internalized, processed, and presented on MHC-I, which is critical to engage antigen-specific CD8^+^ T cells [13,14,15]. Thus, nanoparticle delivery systems enhance antigen cross-presentation, promote co-delivery of both antigen and adjuvants, and therefore improve adjuvant safety, which is ideal for cancer vaccine development. Nonetheless, while robust, many of these nanoparticle vaccines lack consistency in particle size and/or loading of peptide and adjuvant [16]. In addition, some may have some toxicity associated with nanomaterials, all of which compromise the application of these nanoparticle vaccines.

On the other hand, DNA and RNA origami are nucleic acid nanostructures that are programmed to assemble into pre-designed shapes and sizes; therefore, they ensure consistency in shape, size, and molecule incorporation [16,17,18]. Furthermore, we have previously shown that double-stranded RNA (dsRNA) RNA origami (RNA-OG) nanostructures function as adjuvants by acting as toll-like receptor 3 (TLR3) agonists and therefore induce potent innate immune responses that promote adaptive immunity, resulting in tumor regression in tumor-bearing mice [19]. Taking advantage of this platform, we used this self-adjuvanted RNA-OG nanostructure to design a peptide nanovaccine by covalently conjugating tumor peptides onto RNA-OG [20]. While the RNA-OG-peptide nanovaccine induced robust anti-tumor immunity in mice, the amount of peptide loaded into the vaccine was limited (<1 μg per treatment). Thus, to increase the peptide loading, we explored other ways to construct RNA-OG peptide vaccines.

Instead of being covalently linked to the peptides, negatively charged RNA-OG can be complexed to lysine-extended, positively charged peptides through physical interactions, which enables tunable peptide loading. Interestingly, we found that a greater number of peptides complexed with RNA-OG did not induce CD8^+^ T cell proliferation in vitro and failed to generate protective immunity in tumor-bearing mice, which is likely attributed to an ineffective induction of T cell response. However, by lowering the dosage of the peptides complexed to RNA-OG, these nanovaccines improved anti-tumor immunity. Furthermore, stable, covalently assembled RNA-OG-peptide vaccines displayed the best efficacy. These findings show that peptide dosage and complex stability of RNA-OG peptide nanovaccines are critical for vaccine efficacy and the development of effective anti-tumor immunotherapies.

## 2. Materials and Methods

### 2.1. Peptide Sequences

Ovalbumin peptide OVA_257–264_ (pOVA; SIINFEKL) (MW: 963.2 g/mol) (Invivogen, San Diego, CA, USA).

Polylysine-linked pOVA (pOVA-K10; SIINFEKLKKKKKKKKKK) (MW: 2244.9 g/mol) (United Biosystems, Herndon, VA, USA).

FITC-pOVA-K10 peptide (FITC-Ahx-SIINFEKLKKKKKKKKKK) (MW: 2747.4 g/mol) (United Biosystems).

Azide-linked pOVA (pOVA-Az; K(N_3_)-SIINFEKL) (MW: 1115 g/mol).

### 2.2. RNA-OG and RNA-OG-DBCO Generation

RNA-OG is a self-assembling, rectangular RNA nanostructure that was designed, cloned, and generated via in vitro transcription as previously described [19,21]. Briefly, RNA was transcribed from a DNA template (sequence previously provided [19]) and then purified. RNA-OG was self-assembled in 1× PBS from 65 to 25 °C at a ramp of −1 °C per 15 min.

RNA-OG-DBCO was generated for the covalent assembly of peptides as previously described [20]. Briefly, the procedure for generating RNA-OG-DBCO was modified from RNA-OG by using amine-UTP (TriLink BioTechnologies, San Diego, CA, USA) during in vitro transcription. Following self-assembly, the RNA nanostructure was mixed with DBCO-Sulfo-NHS-ester (Click Chemistry Tools, Scottsdale, AZ, USA) and then purified.

### 2.3. Assembly of RNA-OG Peptide Complexes

RNA-OG (1 μM) was mixed with pOVA-K10 or FITC-pOVA-K10 (13–500 μM) for a molar ratio of 1 RNA-OG:13–500 pOVA in PBS and incubated at room temperature (RT) for 30 min. Covalently linked RNA-OG-pOVA was assembled as previously described [20]. Briefly, RNA-OG-DBCO was reacted with pOVA-Az overnight and then dialyzed to generate RNA-OG-pOVA.

### 2.4. Agarose Gel Electrophoresis

For visualization of the complexes, 1 μg RNA-OG, RNA-OG/pOVA-K10, RNA-OG/FITC-pOVA-K10, or RNA-OG-pOVA at specified ratios were run on a 1% agarose gel in 0.5× TBE. Gels were imaged for FITC fluorescence and subsequently stained with SYBR Gold (Invitrogen, Waltham, MA, USA) for RNA detection.

### 2.5. Stability of RNA-OG

RNA stability in serum was tested by supplementing 1 μg RNA-OG or RNA-OG/pOVA-K10 with 10% mouse serum in 10 μL (100 μg/mL RNA). Samples were incubated at 37 °C for 4–24 h and then placed on ice to prevent further degradation. RNase I digestion was performed by incubating 1 μg RNA-OG, RNA-OG/pOVA-K10 (1:13 or 100), or RNA-OG-pOVA with 5U RNase I (ThermoFisher, Waltham, MA, USA) in 10 μL PBS (1 μg RNA:5U RNase I) at RT for 0.5–2 h and then placed on ice to stop digestion.

### 2.6. Atomic Force Microscopy (AFM)

5 μL of each 50 nM sample was deposited on a cleaved mica surface (Ted Pella) and left alone for 30 s. Then, 40 μL of 1× TAE Mg^2+^ buffer and 5 μL of 100 mM NiCl_2_ solution were added to the mica. The sample was subsequently imaged by MultiMode 8 AFM (Bruker, Billerica, MA, USA) in “ScanAsyst in fluid” mode.

### 2.7. Bone Marrow-Derived Dendritic Cell (BMDC) Generation

BMDCs were generated as previously described [20]. Tibias and femurs isolated from 8–12-week-old, naïve C57BL/6 female mice were flushed with RPMI/3%FBS and extracted bone marrow cells were filtered through a 40 μm cell strainer (Biologix, Camarillo, CA, USA). Red blood cells (RBCs) were removed using RBC lysis buffer (BioLegend, San Diego, CA, USA) according to the manufacturer’s protocol. Cells were then washed with PBS+3%FBS and resuspended to 1.5 × 10^6^ cells/mL in RPMI/CM (RPMI-1640 (Sigma-Aldrich, St. Louis, MO, USA) supplemented with 10% heat-inactivated FBS (Gibco, Waltham, MA, USA), 2 mM glutamine, 100 U/mL penicillin (Sigma-Aldrich, St. Louis, MO, USA), 100 μg/mL streptomycin (Sigma-Aldrich), and 50 μM 2ME (Sigma-Aldrich, St. Louis, MO, USA)) + HEPES (10 mM) (Sigma-Aldrich, St. Louis, MO, USA) + L-glutamine (2 mM) (Sigma-Aldrich) + MEM non-essential amino acid (1x) (Gibco, Waltham, MA, USA) (primary cell culture media) + 100 ng/mL Flt3L (Tonbo, Fremont, CA, USA) and then seeded on suspension cell culture dishes. Cells were cultured for a total of 9 days at 37 °C, 5% CO_2_. On day 5, additional Flt3L (100 ng/mL) containing media was added. On day 9, BMDCs were harvested.

### 2.8. BMDC Ex Vivo Stimulation and Antigen Presentation

BMDCs were stimulated as previously described [20]. After harvesting, BMDCs were seeded at 5 × 10^5^ cells/well in a 12-well plate for a density of 5 × 10^5^ cells/mL in primary cell culture media + PMB (25 μg/mL). Cells were then treated with PBS, pOVA-K10 (5 μM), RNA-OG (0.05 μM, 33 μg/mL), RNA-OG (0.05 μM) + pOVA (5 μM), RNA-OG-pOVA (0.05 μM:~0.65 μM), or RNA-OG/pOVA-K10 (0.05 μM:5 μM) and incubated for 18–20 h at 37 °C. pOVA (5 μM) was added during the last 2 h of incubation as a peptide pulse positive control. After stimulation, cells were gently washed with RPMI/CM (3x) and co-cultured with OT-I splenocytes or were harvested, washed, and stained using anti-H2-K^b^/pOVA (BioLegend, San Diego, CA, USA) and anti-CD86 (Tonbo, Fremont, CA, USA) fluorescent antibodies. Stained cells were analyzed via flow cytometry, and the MFI for each treatment was normalized against the positive controls.

### 2.9. Splenocyte Isolation

Spleens were removed from naïve mice, and splenocytes were pushed out using an L-shaped glass rod. Cells were then homogenized with a 22-G syringe and filtered through a 40 μm cell strainer (Biologix, Camarillo, CA, USA). Single-cell suspensions were washed, and RBCs were removed using RBC lysis buffer (BioLegend, San Diego, CA, USA) according to the manufacturer’s protocol.

### 2.10. OT-I T Cell Ex Vivo Proliferation Assay

Splenocytes were isolated from 8–12-week-old naïve OT-I mice according to the protocol above. Splenocytes were stained with Cytopainter proliferation dye (Abcam, Waltham, MA, USA) in Hank’s Balanced Salt Solution (HBSS) (Sigma-Aldrich, St. Louis, MO, USA) (1 × 10^6^ cells/mL) for 30 min at 37 °C. After washing, stained splenocytes were added to treated and washed BMDCs (protocol above) at a ratio of 1 BMDC:20 OT-I splenocytes in primary cell culture media at 37 °C. OT-I splenocytes treated with Concanavalin A (Con A) (10 μg/mL) or PBS without BMDCs were used as controls. After 72 h, cells were harvested and analyzed for T cell proliferation using flow cytometry. Proliferation for each treatment was normalized against the Con A control.

### 2.11. Mice

Female C57BL/6 mice were purchased from Charles River Laboratories. C57BL/6-Tg (TcraTcrb)1100Mjb/J (OT-I) mice (expressing transgenic OVA_257–264_-specific CD8^+^ T cells) were purchased from Jackson Laboratories and bred in-house. The mice were housed in pathogen-free conditions at Arizona State University and were handled in accordance with the Animal Welfare Act and the Arizona State University Institutional Animal Care and Use Committee (IACUC).

### 2.12. Tumor Challenge and Treatments

B16-OVA, a melanoma tumor cell line expressing OVA, was a generous gift from Dr. Richard G. Vile (Mayo Clinic in Rochester). In the therapeutic studies, 6–10-week-old, naïve female C57BL/6 mice were engrafted with B16-OVA (2 or 5 × 10^5^, s.c., right flank). Tumor-bearing mice were treated (5, 10, and 15 days later) with PBS, RNA-OG (40 μg) + pOVA (0.8 μg or 5.8 μg) (1:13 or 100 molar ratio) mixture, RNA-OG (40 μg)/pOVA-K10 (1.8 μg, 14 μg, or 24 μg) (1:13, 100, or 175 molar ratio) physically assembled complex, or RNA-OG-pOVA (40 μg:~0.9 μg) (1:~13 molar ratio) covalent complex (s.c., right flank). Tumor size was measured 2–3 times per week using calipers, and tumor volume (mm^3^) was calculated as (width)^2^ × (length/2). Mice were euthanized at endpoint, determined by tumor size, in accordance with IACUC protocols.

### 2.13. TILs Analysis

A 6–10-week-old, naïve female C57BL/6 mouse adoptively transferred with OT-I splenocytes (10^7^, i.p.) was engrafted 3 days later with B16-OVA (3 × 10^6^, s.c., right flank). When tumors reached 20–80 mm^3^ (11–13 days after tumor inoculation), mice were treated with PBS, RNA-OG (40 μg)/pOVA-K10 (1.8 μg or 24 μg) (1:13 or 175 molar ratio) physically assembled complexes, or RNA-OG-pOVA (40 μg:~0.9 μg) (1:~13 molar ratio) covalent complexes (s.c., right flank). When tumors reached 100–500 mm^3^ (5–7 days post treatment), mice were euthanized, and tumors were harvested.

### 2.14. Tumor Processing

Tumor tissues were isolated and minced with scissors until 1–2 mm in size. The minced tissues were then digested with Liberase LT (50 μg/mL) (Roche, Indianapolis, IN, USA) and DNase (100 μg/mL) for one hour at 37 °C, resuspended in buffer containing EDTA (2 mM) to inactivate the enzymes, and then strained through a 40 μm cell strainer. Cells were washed and resuspended in primary cell culture media. Tumor cells were further purified by centrifuging with Histopaque 1083 (Millipore Sigma, Burlington, MA, USA) to eliminate dead tumor cells following the manufacturer’s protocols. Cells were washed, and then CD45^+^ leukocytes were isolated with magnetic-activated cell sorting (MACS) using CD45 microbeads (Miltenyi Biotech, San Diego, CA, USA) according to the manufacturer’s protocols.

### 2.15. Flow Cytometry

Cells were stained as previously described [20]. Harvested cells were washed with PBS and stained with viability dyes—Ghost Red 780 (Tonbo, Fremont, CA, USA) or Zombie Yellow (BioLegend, San Diego, CA, USA) in PBS for 20 min on ice. Cells were then washed with PBS supplemented with 2% BSA and 0.1% sodium azide (staining buffer) and incubated with anti-mouse CD16/32 antibody (FcR blocker) (BioLegend, San Diego, CA, USA) and antibody staining mixes for 20 min on ice. Staining mixes included fluorescent antibodies specific to H2-K^b^/pOVA, CD86, CD45, CD11c, I-A/I-E (MHC-II), CD11b, B220, CD3e, CD8a, CD4, PD-1, and PD-L1 (BioLegend (San Diego, CA, USA), Tonbo (Fremont, CA, USA), and ThermoFisher (Waltham, MA, USA)). When H2-K^b^-pOVA dextramer-PE (Immudex, Copenhagen, Denmark) was used, it was added to cells for 10 min prior to antibody staining mixes following the manufacturer’s protocols. After cell surface staining, cells were washed with staining buffer and proceeded to nuclear staining or fluorescence quantification using the Attune NxT Flow Cytometer (ThermoFisher, Waltham, MA, USA).

Nuclear staining. Cells were fixed with transcription factor fix/perm solution (Tonbo, Fremont, CA, USA) for 20 min on ice. Fixed cells were then permeabilized using flow cytometry perm buffer (Tonbo) supplemented with 2% FBS for 20 min on ice. Next, permeabilized cells were incubated with FoxP3 fluorescent antibody (BioLegend) for 20 min on ice. After staining, cells were washed, and fluorescence was quantified using the Attune NxT Flow Cytometer (ThermoFisher, Waltham, MA, USA). Gating and fluorescence were analyzed using FlowJo v7.

### 2.16. Statistical Analysis

Statistical analyses were performed using GraphPad Prism v10. The values in the graphs ARE shown as mean ± standard deviation. To compare multiple groups, we used one-way ANOVA followed by Sidak’s multiple comparisons test. Kaplan–Meier survival curves were assessed using Log-rank tests to compare mouse treatment groups in the therapeutic studies. Significant data were indicated as follows: * *p* < 0.05, ** *p* < 0.01, *** *p* < 0.001, **** *p* < 0.0001.

## 3. Results

### 3.1. RNA-OG Remains Intact Following RNA-OG/Peptide-K10 Assembly

**Figure 1:**
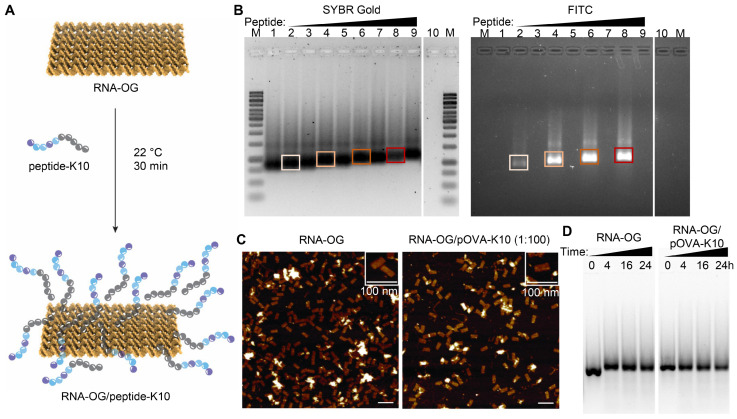
Structure and stability of RNA-OG/peptide-K10 complex. (**A**) Schematic of RNA-OG/pOVA-K10 peptide assembly from RNA-OG and polylysine-linked peptides (peptide-K10). (**B**) Agarose gel electrophoresis analysis of RNA-OG/pOVA-K10 at different ratios. SYBR Gold staining (**left**) and FITC fluorescence imaging (**right**) of 1. RNA-OG; 2–9. RNA-OG/pOVA-K10 at an RNA-OG:pOVA-K10 ratio of 1:50 (2–3), 1:100 (4–5), 1:150 (6–7), 1:200 (8–9), using either pOVA-K10 (3, 5, 7, 9) or FITC-pOVA-K10 (2, 4, 6, 8); 10. FITC-pOVA-K10. Bands of the corresponding samples are highlighted by the same color box. M denotes 1kb DNA marker. (**C**) AFM imaging of RNA-OG and RNA-OG/pOVA-K10 at a 1:100 ratio. (**D**) Agarose gel electrophoresis analysis of RNA degradation in 10% serum. RNA-OG or RNA-OG/pOVA-K10 (1:100) was incubated in 10% mouse serum for 4, 16, or 24 h at 37 °C.

RNA-OG was evaluated as a peptide assembly platform for peptide vaccine construction. Taking advantage of the negatively charged RNA-OG, we attached positively charged polylysine-linked peptides (peptide-K10) to RNA-OG, forming RNA-OG/peptide-K10 complexes (Figure 1A). In particular, RNA-OG was mixed with ovalbumin OVA_257–264_-K10 peptides (pOVA-K10) at various molar ratios of 1 RNA-OG:50–200 pOVA-K10 to form RNA-OG/pOVA-K10 complexes. Using fluorescein isothiocyanate (FITC)-linked peptides (FITC-pOVA-K10), these complexes were observed on gel electrophoresis by FITC fluorescence and SYBR Gold-staining of RNA (Figure 1B). The co-localization of the RNA bands and FITC signal for RNA-OG/FITC-pOVA-K10 complexes (lanes 2, 4, 6, and 8) depicts the peptides assembled with RNA-OG. Furthermore, RNA-OG (stained by SYBR Gold) and FITC bands noticeably shifted upwards as the amount of peptide increased from 50 to 200 pOVA-K10 (lanes 2–9), indicating tunable loading of peptides onto RNA-OG. Thus, RNA-OG was readily assembled with pOVA-K10 up to a ratio of 1 RNA-OG:200 pOVA-K10. To test the upper limits of this assembly, RNA-OG was mixed with pOVA-K10 at a molar ratio of 1 RNA-OG:100–500 FITC-pOVA-K10 (Figure S1A). At ratios over 1:300, RNA-OG/FITC-pOVA-K10 complexes began to show reduced signal intensity, and the bands displayed upward smears on the gel (lanes 5–6), suggesting aggregation of the complexes. Based on these findings, we estimated that each RNA-OG might bind up to 300 pOVA-K10 without forming large aggregates. Thus, the physical assembly of peptides to RNA-OG enables a higher amount of peptide loading than the covalent conjugation of peptides to RNA-OG, which is limited to 13 peptides per RNA-OG [20].

To investigate whether peptide addition affects the integrity and stability of RNA-OG nanostructures, the RNA-OG/pOVA-K10 complexes formed at various RNA-OG to pOVA-K10 ratios were examined by both atomic force microscopy (AFM) imaging and gel analysis after being exposed to mouse serum. As shown by AFM, RNA-OG/pOVA-K10 at all tested ratios (1 RNA-OG:13, 100, or 200 pOVA-K10) formed uniform rectangular nanostructures that were identical to peptide-free RNA-OG, indicating that RNA-OG conformation remained intact following peptide assembly (Figure 1C and Figure S1B). Moreover, the RNA-OG/pOVA-K10 complexes formed at a 1:100 ratio of RNA-OG to peptides were evaluated for their stability in 10% serum alongside RNA-OG (Figure 1D). Both RNA-OG and RNA-OG/pOVA-K10 displayed distinct bands even after 24 h incubation with 10% serum, indicating that RNA-OG remained intact for at least 24 h in 10% serum. Thus, these analyses show that the addition of peptides did not significantly alter the structural stability of RNA-OG.

### 3.2. RNA-OG/pOVA-K10 Promotes Robust DC Maturation and Presentation but Minimal T Cell Proliferation

**Figure 2:**
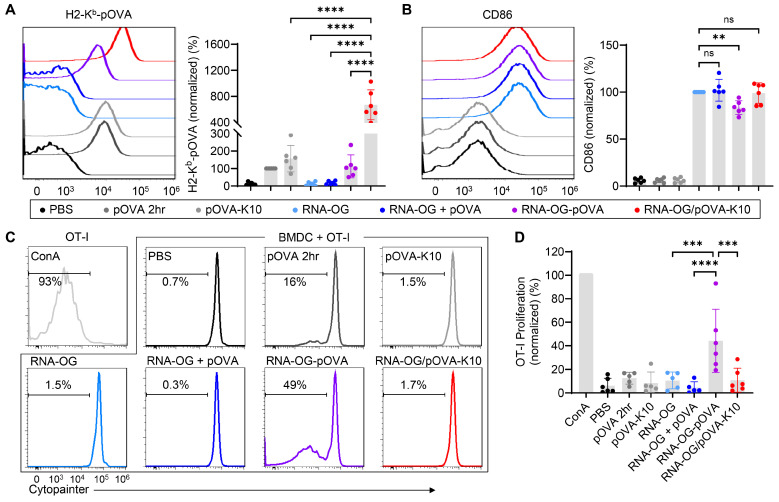
Antigen presentation and stimulation of BMDCs and T cell proliferation. (**A**,**B**) BMDCs treated overnight with PBS, pOVA-K10, RNA-OG, RNA-OG mixed with pOVA (RNA-OG + pOVA), covalently-linked RNA-OG-pOVA, or physically assembled RNA-OG/pOVA-K10 (1:100), or incubated for 2 h with pOVA, were analyzed via flow cytometry, and CD11c^+^ BMDCs were displayed for MHC-I/pOVA presentation (**A**) and CD86 expression (**B**). A representative histogram overlay (**left**) and quantification of the MFI normalized to the positive control–pOVA 2 h (**A**) or RNA-OG (**B**) (**right**) are shown. (**C**,**D**) OT-I splenocytes co-cultured 3 days with treated BMDCs were assessed for proliferation using a cytopainter proliferation dye. OT-I splenocyte incubation with ConA was used as a positive control. CD8^+^ T cell (CD3^+^CD8^+^) proliferation, indicated by the diminishment of cytopainter fluorescence and displayed as representative histograms (**C**), was quantified and normalized to the ConA control (**D**). One-way ANOVA followed by multiple comparisons tests was used for statistical analysis (*n* = 5–6). ** indicates *p* < 0.01, *** indicates *p* < 0.001, and **** indicates *p* < 0.0001.

Successful peptide vaccines are required to induce antigen presentation (signal 1) and co-stimulation (signal 2) in APCs, particularly DCs, to activate T cells [7]. As an intrinsic TLR3 agonist, RNA-OG nanostructure has been shown to function as a potent adjuvant [19]. Here, we evaluated whether RNA-OG/pOVA-K10 could activate bone marrow-derived DCs (BMDCs) and promote antigen presentation. Specifically, BMDCs were treated overnight with RNA-OG/pOVA-K10 or equivalent amounts of RNA-OG with or without pOVA (ovalbumin peptide, OVA_257–264_) and then examined for their antigen presentation by using fluorescence-labeled antibodies specific to MHC-I/pOVA to stain DCs for flow cytometry analysis. Two-hour pulsing with pOVA, representing direct binding of pOVA to MHC-I, served as a positive control. As seen in Figure 2A, the RNA-OG/pOVA-K10 complex conferred elevated levels of peptide presentation in BMDCs, much higher than that of the mixture of RNA-OG + pOVA, showing that peptides in the complex are better presented than the mixture of RNA-OG and pOVA. Notably, treatment with pOVA-K10 alone also resulted in higher levels of peptide presentation than the RNA-OG + pOVA mixture, indicating that the additional 10 lysine residues on the peptide somehow increase the MHC-I/pOVA presentation, potentially either by increasing the peptide stability for subsequent binding to MHC-I or promoting pOVA-K10 internalization for cross-presentation onto MHC-I. Nonetheless, RNA-OG/pOVA-K10 complexes resulted in a 4-fold increase in peptide presentation over the pOVA-K10 alone. Thus, pOVA-K10 peptides assembled with RNA-OG are highly effective for peptide presentation by BMDCs, presumably via RNA-OG-mediated cellular uptake followed by cross-presentation to MHC-I. Furthermore, all RNA-OG-containing treatments promoted BMDC maturation, indicated by an increased expression of CD86 (Figure 2B). Thus, RNA-OG/pOVA-K10 complexes were able to induce DCs to present both signals 1 and 2.

These two signals are critical to the initiation of a CD8^+^ T cell response. To determine if BMDCs treated with RNA-OG/pOVA-K10 could activate CD8^+^ T cells, we used a co-culture assay of treated BMDCs and splenocytes isolated from an OT-I mouse model, which expresses a transgene encoding a pOVA-specific T cell receptor (TCR_pOVA_), resulting in almost all CD8^+^ T cells bearing TCR_pOVA_ in OT-I mice. BMDCs under various treatments described above were co-cultured with Cytopainter labeled OT-I splenocytes and assessed for T cell proliferation, in which mitogen concanavalin A (Con A) was used as a positive control. To our surprise, despite a high level of cross-presentation of RNA-OG/pOVA-K10 by BMDCs, co-cultured CD8^+^ T cells showed little or no proliferation, although RNA-OG-pOVA (pOVA assembled via covalent linkage) showed elevated levels of proliferation (Figure 2C,D). One possible explanation is that RNA-OG/pOVA-K10 may not be sufficiently stable for sustained peptide cross-presentation to activate OT-I T cells. Instead, free pOVA released from RNA-OG/pOVA-K10 during the co-culture might have directly bound to any MHC-I^+^ cells without co-stimulators, thereby resulting in anergy.

Although RNA-OG/pOVA-K10 showed good stability in 10% serum (Figure 1D) or upon RNase treatment using previously described conditions (i.e., 1 U of RNase I with 1 μg RNA-OG for 20 min) [19], they may not tolerate a higher level of nuclease treatment. To further delineate different vulnerabilities of various RNA-OG peptide complexes to RNase treatment, we examined these RNA-OG-based peptide complexes under a 5-fold higher concentration of the RNase for a longer incubation time, i.e., 5 U RNase I per 1 μg RNA-OG, for 0.5–2 h to examine how the complexation of peptide-K10 to RNA-OG at various ratios (1 RNA-OG:0, 13, 100 pOVA-K10) impacts the stability of RNA-OG (Figure S2A). As shown by the intensity of the RNA-OG band, RNA-OG displayed only minor degradation after 2 h in RNase (lane 4), whereas RNA-OG/pOVA-K10 (1:100) was degraded by 1 h and completely gone by 2 h (lanes 11 and 12, respectively). In comparison, RNA-OG/pOVA-K10 (1:13) was somewhat resistant to RNase as the RNA-OG band remained intact at 1 h (lane 7) and was still visible at 2 h (lane 8). On the other hand, the covalently assembled complex (RNA-OG-pOVA) remained intact even after 2 h of RNase treatment (lane 16), presenting the highest stability among various complexes. Thus, the more pOVA-K10 peptides assembled to RNA-OG, the less stable the RNA-OG/pOVA-K10 complexes became. As a result, the free peptides released from degraded RNA-OG might have subsequently compromised CD8^+^ T cell activation. Taken together, our findings showed that while RNA-OG/pOVA-K10 with a high peptide load induced peptide presentation and co-stimulation in BMDCs, they failed to properly engage CD8^+^ T cells for their activation and proliferation. This finding indicates that peptide presentation by BMDCs does not inherently lead to T cell proliferation.

### 3.3. Increased Peptide Amount in RNA-OG/pOVA-K10 Reduces Therapeutic Efficacy

**Figure 3:**
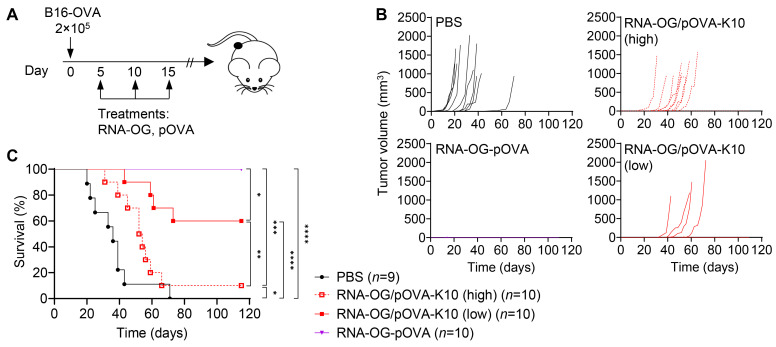
Therapeutic efficacy of RNA-OG/pOVA-K10 (low or high peptide) vaccines. (**A**) Treatment schedule. Mice engrafted with 2 × 10^5^ B16-OVA (s.c., flank) were treated with PBS, physically assembled RNA-OG/pOVA-K10 (1:13 or 175), or covalently linked RNA-OG-pOVA (1:~13) on days 5, 10, and 15 (s.c., flank) after tumor inoculation. (**B**) Tumor growth and (**C**) survival of mice following tumor administration were monitored. Data is compiled from two replicate experiments (*n* = 9–10). Kaplan–Meier statistical analysis was used to compare each treatment group. * Indicates *p* < 0.05, ** indicates *p* < 0.01, *** indicates *p* < 0.001, and **** indicates *p* < 0.0001.

To investigate whether RNA-OG/pOVA-K10 complexes could elicit an anti-tumor response, we tested this vaccine in vivo. Using B16-OVA, a melanoma tumor model expressing OVA, we evaluated these RNA-OG peptide complexes, containing ‘low’ (1 RNA-OG:13 pOVA-K10) or ‘high’ (1 RNA-OG:≥100 pOVA-K10) peptide amounts (see Table 1), for their induction of anti-tumor immunity. B16-OVA tumor-bearing mice were treated with RNA-OG/pOVA-K10_low_, RNA-OG/pOVA-K10_high_, or covalently linked RNA-OG-pOVA three times starting on day 5 after tumor inoculation (Figure 3A). The treatments with RNA-OG/pOVA-K10_low_ or RNA-OG-pOVA delayed or eliminated tumor growth, while the treatment with RNA-OG/pOVA-K10_high_ only resulted in a slight delay in tumor growth compared to the control PBS group (Figure 3B). The difference was highlighted by the increased survival of mice treated with RNA-OG/pOVA-K10_low_ (6/10 mice survived) compared to those treated with RNA-OG/pOVA-K10_high_ (1/10 mice survived) (Figure 3C). In contrast, both dosages of the mixture of RNA-OG + pOVA showed similar levels of survival, although mice treated with RNA-OG + pOVA_low_ had delayed tumor growth compared to those treated with RNA-OG + pOVA_high_ (Figure S3). On the other hand, the mice treated with RNA-OG-pOVA, with a low number of peptides covalently linked to RNA-OG, had the greatest survival, even better than RNA-OG/pOVA-K10_low_, suggesting that both a low peptide amount and possibly stable (covalent) linkage may render the best anti-tumor immunity (Figure 3C).

In a similar study, mice bearing larger tumor loads (5 × 10^5^ B16-OVA) experienced slightly better survival when treated with RNA-OG/pOVA-K10_low_ and even greater with RNA-OG-pOVA compared to RNA-OG/pOVA-K10_high_ (Figure S4). With this high tumor load, covalently linked RNA-OG-pOVA was found again to induce the most effective immunity, resulting in >50% survival of the mice. For physically assembled RNA-OG/pOVA-K10 vaccines, the difference between RNA-OG/pOVA-K10_low_ and RNA-OG/pOVA-K10_high_ was less apparent at this high tumor load; nonetheless, lowering the amount of peptide loaded onto RNA-OG moderately improved protective immunity. Thus, peptide dosage and complex stability of the vaccines affected the anti-tumor immunity generated in mice.

### 3.4. Failed Immunity Initiated at the Tumor Site Attributed to an Early Induction of T Cell Exhaustion

**Figure 4:**
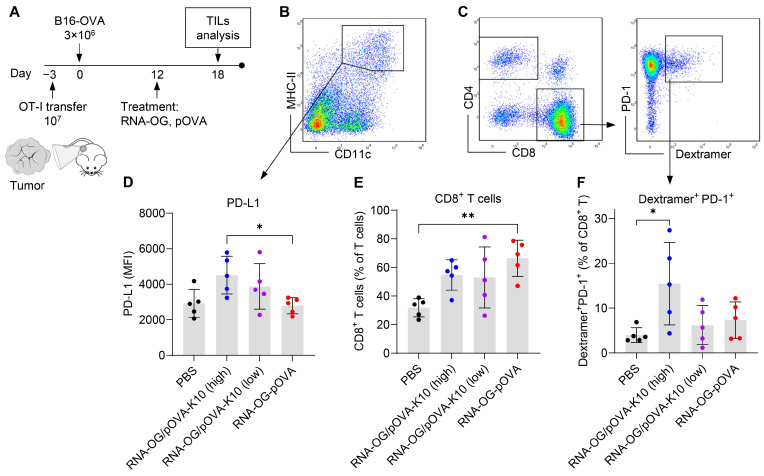
TILs assessment following treatment with RNA-OG peptide vaccines. (**A**) Experiment schedule. Mice adoptively transferred with OT-I splenocytes (10^7^, i.p.) were engrafted with B16-OVA tumors (3 × 10^6^, s.c.) and then treated with PBS, RNA-OG/pOVA-K10_high_, RNA-OG/pOVA-K10_low_, or RNA-OG-pOVA (s.c.). Five to seven days later, tumors were isolated, and TILs were analyzed via flow cytometry. (**B**,**C**) Representative flow cytometry gating of (**B**) DCs from CD45^+^ leukocytes and (**C**) T cell distribution among CD3^+^ T cells in the tumor. Pseudocolor density plot where red represents high cell density and blue represents low cell density. (**D**) PD-L1 expression in MHC-II^+^ DCs. (**E**) Distribution of CD8^+^ T cells. (**F**) Percentage of H2-K^b^/pOVA dextramer^+^PD-1^+^ CD8^+^ T cells. One-way ANOVA with multiple comparisons was used for statistical analysis (*n* = 5). * Indicates *p* < 0.05 and ** indicates *p* < 0.01.

We were intrigued by the poor protective immunity generated by the RNA-OG/pOVA-K10_high_ vaccine (Figure 3) and speculated that this vaccination might fail to elicit sustained immunity, e.g., by inducing CD8^+^ T cell exhaustion. It is known that upon their activation, T cells upregulate their PD-1 expression, which is an indicator of a high level of T cell activation and exhaustion [22]. PD-L1 expression by tumor cells and APCs upon activation can suppress T cell activity via PD-L1/PD-1 engagement [23]. To examine these immune cells during the early stages of T cell activation, we analyzed tumor-infiltrating leukocytes (TILs) after a single vaccination in B16-OVA tumor-bearing mice engrafted with pOVA-specific CD8^+^ T cells. Specifically, OT-I splenocytes were adoptively transferred prior to B16-OVA tumor engraftment. Once the tumor mass reached a palpable size (20–80 mm^3^, ranging from 11 to 13 days after tumor cell injection), mice were randomly assigned for a single treatment with RNA-OG/pOVA-K10_high_, RNA-OG/pOVA-K10_low_, or RNA-OG-pOVA vaccines. Seven days later, tumor masses were collected for TIL analysis (for similar sizes upon isolation, tumors that grew faster were harvested 5 days after treatment) (Figure 4A). Different types of immune cells, including MHC-II^+^ DCs (MHC-II^+^CD11c^+^), CD8^+^ T cells (CD3^+^CD8^+^), and CD4^+^ T cells (CD3^+^CD4^+^), were gated according to the flow cytometry diagrams in Figure 4B,C. We analyzed tumor-infiltrating DCs and found that MHC-II^+^ DCs from mice treated with RNA-OG/pOVA-K10_high_ displayed elevated levels of PD-L1 compared to those treated with RNA-OG-pOVA, suggesting that these DCs may be more adept at suppressing T cells via the PD-L1/PD-1 axis (Figure 4D). Similarly, the DCs of mice treated with RNA-OG/pOVA-K10_low_ also displayed somewhat high levels of PD-L1. Thus, administration of physically assembled complexes, i.e., RNA-OG/pOVA-K10_high_ and RNA-OG/pOVA-K10_low_, at the tumor site increased the number of DCs that have immunosuppressive potential, which was not seen in the vaccination with covalently conjugated RNA-OG-pOVA complexes. On the other hand, a higher number of CD8^+^ T cells was found in the tumors of the mice treated with RNA-OG-pOVA than in the PBS control (Figure 4E). Interestingly, when examining the functional activity of pOVA-specific OT-I T cells using MHC-I/pOVA dextramer staining, we found that RNA-OG/pOVA-K10_high_ treatment resulted in an elevation of dextramer^+^/PD-1^+^ CD8^+^ T cells, which was not observed in the mice treated with either RNA-OG/pOVA-K10_low_ or RNA-OG-pOVA vaccines (Figure 4F). Thus, a single vaccination of RNA-OG/pOVA-K10_high_ led to early T cell exhaustion. Therefore, TILs from mice vaccinated with the RNA-OG/pOVA-K10_high_ vaccine may develop poor immunity by both upregulating PD-L1 in DCs and PD-1 in tumor-specific CD8^+^ T cells early on, leading to T cell exhaustion. On the other hand, RNA-OG/pOVA-K10_low_ may not induce the same level of exhaustion, as it did not increase PD-1 in tumor-specific CD8^+^ T cells, despite increased levels of PD-L1 in DCs. Furthermore, RNA-OG-pOVA promotes an effective CD8^+^ T cell response without inflicting suppression, which was reflected in a greater trend of the CD8^+^:CD4^+^ T cell ratio as well as the CD8^+^:regulatory T cell (T_reg_) (CD3^+^CD4^+^Foxp3^+^) ratio compared to the other treatment groups (Figure S5B,C). This finding suggests that low levels of peptide in the RNA-OG peptide complex might induce a balanced level of CD8^+^ T cell activation without inflicting suppression that leads to failed immunity. Thus, greater amounts of peptide in the RNA-OG/pOVA-K10 complex could have induced early T cell exhaustion, which may explain the lack of anti-tumor immunity developed in tumor-bearing mice.

## 4. Discussion

In this study we evaluated the immunogenicity and therapeutic efficacy of several RNA-OG peptide vaccines against tumor growth. They were based on a self-adjuvanted RNA-OG nanostructure assembled physically with different amounts of antigenic peptides extended with 10 lysine residues. This physical assembly is simple and tunable in terms of the number of peptides for attaching to RNA-OG, i.e., 10–200 peptide molecules per RNA-OG molecule (Figure 1B), which is much higher than the previously reported RNA-OG-peptide nanovaccine that uses covalent linkage to assemble a maximum of 13 peptides per RNA-OG [20].

However, to our surprise, we found that the RNA-OG/peptide-K10 with a low level of peptide, i.e., RNA-OG/peptide-K10_low_, resulted in better protective anti-tumor immunity than the RNA-OG/peptide-K10 with a high peptide load, i.e., RNA-OG/pOVA-K10_high_ (Figure 3). Interestingly, in our controls of RNA-OG mixed with pOVA, vaccination with the low amount of peptide also showed a slight delay in tumor growth compared to the one with the high amount in the same setting (Figures S3B and S4B), reflecting better immunity elicited by the low peptide dosage. Our finding is in line with a previous report that high peptide dosage in the vaccine could reduce tumor immunity, which was attributed to the deletion of the peptide-specific CD8^+^ T cells [24].

As shown in Figure S2A, the high amount of peptide-K10 loaded to RNA-OG resulted in decreased stability of the RNA-OG/pOVA-K10_high_ complex. It is possible that this high peptide loading may render the RNA-OG/pOVA-K10 vaccine less stable in vivo, leading to degradation of the vaccine complexes and/or dissociation of pOVA-K10 peptides from RNA-OG and subsequent peptide degradation, both of which may prevent antigen cross-presentation by DCs to CD8^+^ T cells. On the other hand, a large amount of released (and possibly cleaved) pOVA peptides may directly bind to MHC-I on the surface of any cell, including non-APCs that do not express co-stimulatory signals, such that they could lead to T cell anergy [14,25], analogous to the poor immunity induced by a high dosage of peptide vaccines reported previously [24]. In tumor-bearing mice, treatment with RNA-OG/pOVA-K10_high_, but not the other two forms of RNA-OG assembled complexes, was found to be associated with an early upregulation of PD-1 on pOVA-specific T cells (Figure 4). Putting all this data together, we suggest that high doses of peptides in RNA-OG/peptide-K10 nanovaccines, presumably owing to their intrinsic instability and therefore high levels of free pOVA peptides, failed to mount effective tumor-specific T cell immunity. Thus, dosage and stability of RNA-OG peptide nanovaccines are critical to the efficacy of anti-tumor T cell immunity.

While the covalently linked RNA-OG-pOVA vaccine could elicit strong protective immunity, it is limited by peptide loading, which hinders incorporation and optimization of additional antigenic epitopes. By using polylysine-linked peptides to complex with RNA-OG, we can include multiple neoantigen epitopes with controlled amounts. However, to take advantage of this strategy for generating effective and multi-specific immunity against tumor cells, we need to determine an effective peptide load that will not destabilize RNA-OG nanostructures in vivo. The RNase I-based degradation assay (Figure S2A) was used as a representative test to assess the structural stability of RNA-OG/pOVA-K10. This assay, however, might not have recapitulated the in vivo situation or could have overestimated its instability. Further titration and evaluation of peptide-to-RNA-OG ratios should be conducted to identify a threshold dosage of peptides such that the complex can be retained long enough to confer the immunogenicity of the assembled peptides. A low peptide number assembled on RNA-OG would induce a peptide-specific T cell response without triggering exhaustion. Then, multiple peptides bearing different antigenic epitopes could be physically attached to RNA-OG, so that multiple T cell clones can be activated without undergoing exhaustion. Moreover, we can use this simple peptide-K10 strategy together with the covalent linkage to assemble both MHC-I and MHC-II epitopes or longer synthetic peptides to further enhance vaccine immunity, as reported by other groups [25,26,27,28]. Thus, this work not only improves our understanding of RNA-OG as a nanovaccine assembly platform but also presents a strategy to optimize vaccine efficacy, which aids in the design of robust peptide vaccines for translational cancer immunotherapy.

## Figures and Tables

**Table 1:** Nomenclature, assembly strategy, and dosage of RNA-OG peptide vaccines.

Name	Assembly Strategy	Molar Ratio (RNA-OG:Peptide)	Vaccine Dosage (RNA-OG:pOVA Peptide)
RNA-OG-peptide	Covalent linkage	1:13	40 μg:0.9 μg
RNA-OG/peptide-K10_low_	Physical assembly	1:13	40 μg:1.8 μg
RNA-OG/peptide-K10_high_	Physical assembly	1:≥100	40 μg:≥14 μg
RNA-OG + peptide_low_	Non-assembled	1:13	40 μg:0.8 μg
RNA-OG + peptide_high_	Non-assembled	1:100	40 μg:5.8 μg

## Data Availability

The original contributions presented in this study are included in the article/Supplementary Material. Further inquiries can be directed to the corresponding authors.

## Supplementary Materials

The following supporting information can be downloaded at: https://www.mdpi.com/article/10.3390/vaccines13060560/s1, Figure S1: Maximum peptide assembly of RNA-OG/pOVA-K10 complex; Figure S2: Stability of RNA-OG pOVA complexes in RNase I; Figure S3: Therapeutic efficacy of mixture RNA-OG + pOVA (low or high peptide) vaccines; Figure S4: Therapeutic efficacy of RNA-OG/pOVA-K10 (low or high peptide) in larger tumors; Figure S5: TILs T cell distribution.

## Abbreviations

The following abbreviations are used in this manuscript:

AFM	Atomic force microscopy
APC	Antigen-presenting cell
BMDC	Bone marrow-derived dendritic cell
ConA	Concanavalin A
DC	Dendritic cell
dsRNA	Double-stranded RNA
FITC	Fluorescein isothiocyanate
HBSS	Hank’s Balanced Salt Solution
IACUC	Institutional Animal Care and Use Committee
i.p.	Intraperitoneal
LN	Lymph node
MACS	Magnetic-activated cell sorting
PAMP	Pathogen-associated molecular pattern
PMB	Polymyxin B
pOVA	Ovalbumin peptide, OVA_257–264_
pOVA-Az	Azide-linked ovalbumin peptide
pOVA-K10	Polylysine-linked ovalbumin peptide
PRR	Pattern recognition receptor
RBC	Red blood cell
RNA-OG	RNA origami
RT	Room temperature
s.c.	Subcutaneous
TCR_pOVA_	pOVA-specific T cell receptor
TIL	Tumor-infiltrating leukocyte
TLR3	Toll-like receptor 3
T_reg_	Regulatory T cell
