# Supplementary material for: Adjuvanted RNA Origami—A Tunable Peptide Assembly Platform for Constructing Cancer Nanovaccines
Source: Vaccines (Basel). 2025 May 25;13(6):560. doi: 10.3390/vaccines13060560 (PMC12197741; doi:10.3390/vaccines13060560)
Supplement: Supplementary file 1 [file vaccines-13-00560-s001.zip › vaccines-3621476-supplementary.pdf]

Adjuvanted RNA Origami, a Tunable Peptide-Assembly Platform, for Constructing Cancer Nanovaccines

Theresa Yip[1,2], Xinyi Tu[2,3], Xiaodong Qi[2,3], Hao Yan[2,3*], Yung Chang[1,2*]

1 School of Life Sciences, Arizona State University, Tempe, AZ 85281, USA.

2 Biodesign Center for Molecular Design and Biomimetics, Biodesign Institute, Arizona State University, Tempe, AZ 85281, USA.

3 School of Molecular Sciences, Arizona State University, Tempe, AZ 85281, USA.

* Correspondence to: Hao.Yan@asu.edu, Yung.Chang@asu.edu

Supplemental Figures

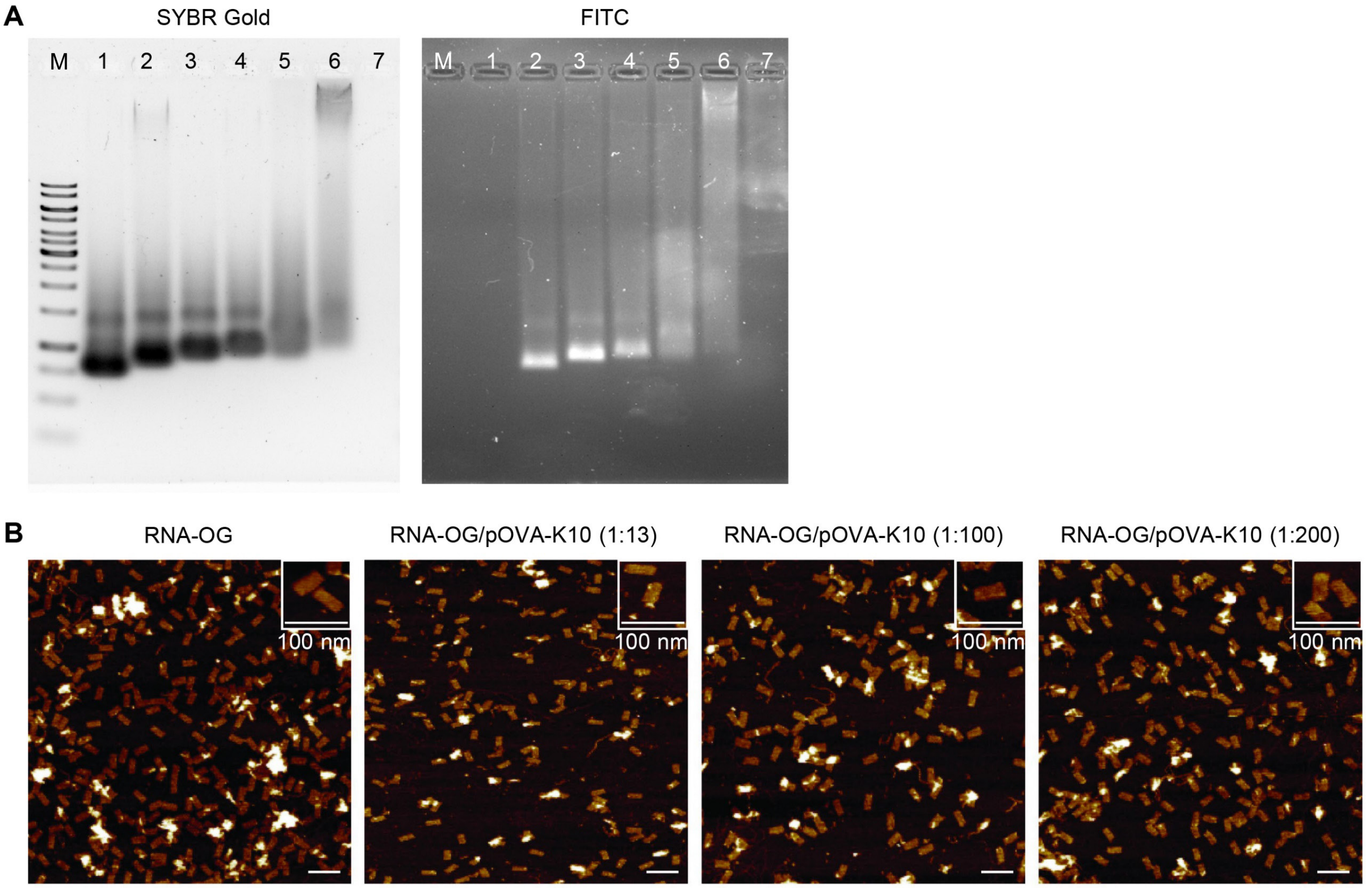

Figure S1. Maximum peptide assembly of RNA-OG/pOVA-K10 complex. A) Agarose gel electrophoresis analysis of RNA-OG/FITC-pOVA-K10 at different ratios. SYBR Gold staining (left) and FITC fluorescence (right) of 1. RNA-OG; 2-6. RNA-OG/pOVA-K10-FITC at an RNA-OG : FITC-pOVA-K10 ratio of 1:100 (2), 1:200 (3), 1:300 (4), 1:400 (5), and 1:500 (6); 7. FITC-pOVA-K10. M denotes 1kb DNA marker. **B)** AFM imaging of RNA-OG and RNA-OG/pOVA-K10 at a 1 RNA-OG : 13, 100, or 200 pOVA-K10 ratio.

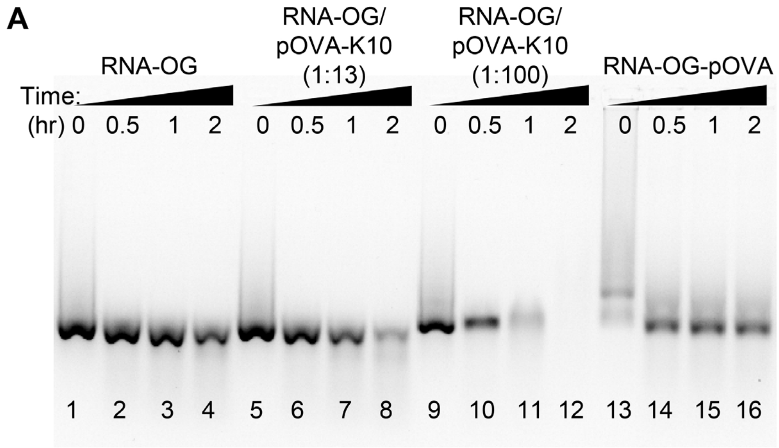

Figure S2. Stability of RNA-OG pOVA complexes in RNase I. A) Agarose gel electrophoresis analysis of RNA degradation in RNase I. 1 μg RNA-OG, RNA-OG/pOVA-K10 (1:13) or (1:100), or RNA-OG-pOVA incubated with RNase I (5U) at room temperature for 0.5, 1, or 2hrs were run on an agarose gel.

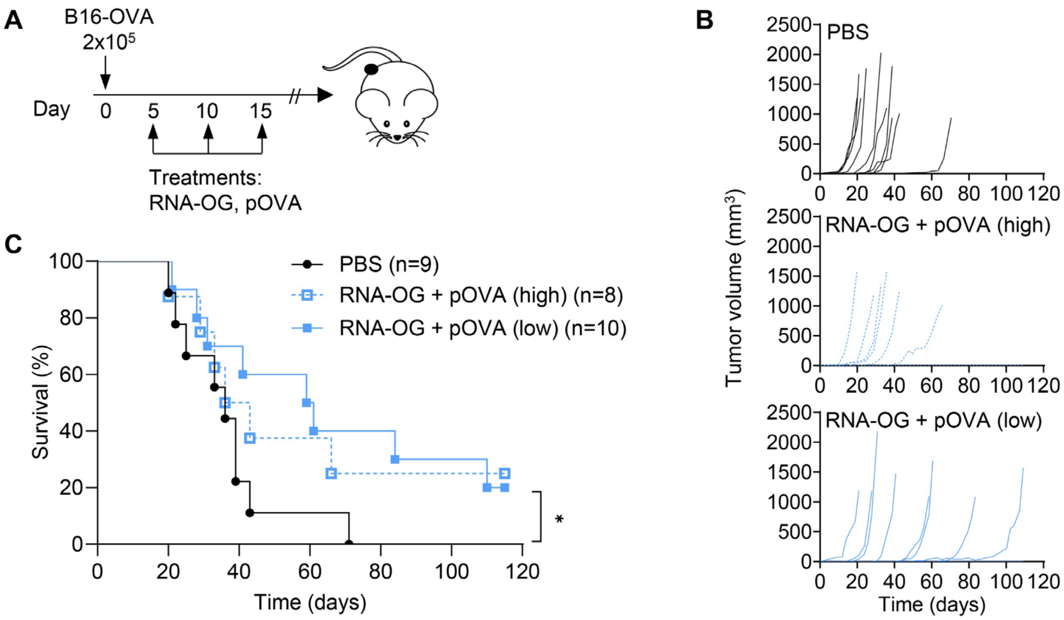

Figure S3. Therapeutic efficacy of mixture RNA-OG + pOVA (low or high peptide) vaccines. A) Treatment schedule. Mice engrafted with 2x10^5 B16-OVA (s.c., flank) were treated with PBS or RNA-OG + pOVA (1:13 or 100) on days 5, 10, and 15 (s.c., flank) after tumor inoculation. **B)** Tumor growth and **C)** Survival of mice following tumor administration was monitored. Data is compiled from two replicate experiments (n=8-10). Kaplan-Meier statistical analysis was used to compare each treatment group. * indicates $p<0.05$.

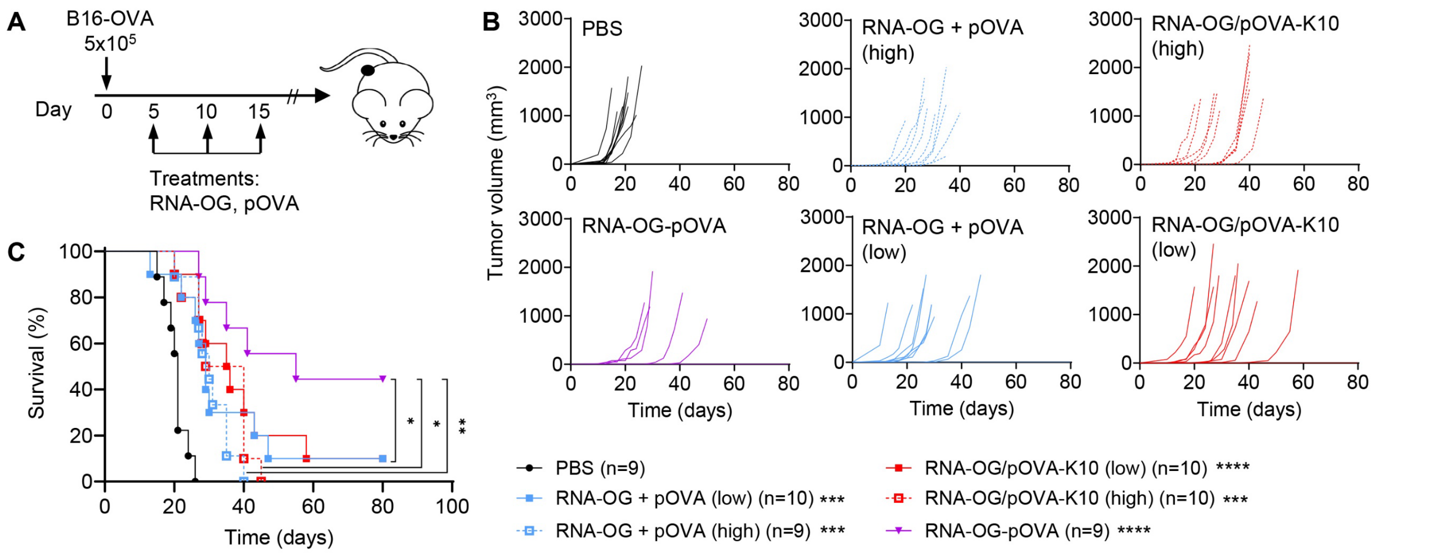

Figure S4. Therapeutic efficacy of RNA-OG/pOVA-K10 (low or high peptide) in larger tumors. A) Treatment schedule. Mice engrafted with $5x10^5$ B16-OVA (s.c., flank) were treated with PBS, RNA-OG + pOVA (1:13 or 1:100), RNA-OG/pOVA-K10 (1:13 or 1:100), RNA-OG-pOVA (1:~13) on days 5, 10, and 15 (s.c., flank) after tumor inoculation. **B)** Tumor growth and **C)** Survival of mice following tumor administration was monitored. Data is compiled from two replicate experiments (n=9-10). Kaplan-Meier statistical analysis was used to compare each treatment group. * indicates p<0.05, ** indicates p<0.01, *** indicates p<0.001, **** indicates p<0.0001. Symbols next to sample name, indicates statistical significance compared to PBS.

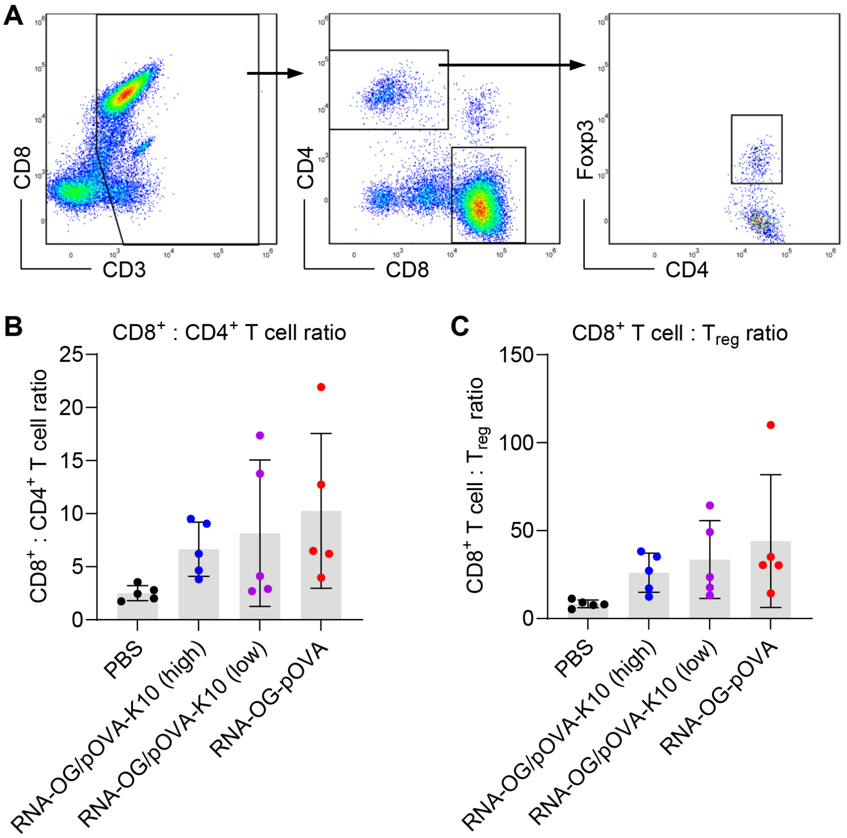

Figure S5. TILs T cell distribution. Following the experiment schedule in Figure 4, TILs were isolated from B16-OVA tumors and assessed via flow cytometry. **A)** Representative flow cytometry gating of $CD8^+$ T cells ($CD3^+CD8^+$), $CD4^+$ T cells ($CD3^+CD4^+$), and T_{regs} ($CD3^+CD4^+Foxp3^+$). **B)** $CD8^+$ T cell : $CD4^+$ T cell ratio among TILs. **C)** $CD8^+$ T cell : T_{reg} ratio among TILs. One-way ANOVA with multiple comparisons were used for statistical analysis (n=5).
